# Evaluation of Band Selection for Spectrum-Aided Visual Enhancer (SAVE) for Esophageal Cancer Detection

**DOI:** 10.7150/jca.102759

**Published:** 2025-01-01

**Authors:** Yen-Chun Chen, Riya Karmakar, Arvind Mukundan, Chien-Wei Huang, Wei-Chun Weng, Hsiang-Chen Wang

**Affiliations:** 1 Department of Gastroenterology, Dalin Tzu Chi Hospital, Buddhist Tzu Chi Medical Foundation, No. 2, Minsheng Road, Dalin, Chiayi, 62247 Taiwan.; 2 School of Medicine, Tzuchi University, No.701, Sec. 3, Zhongyang Rd. Hualien 97004, Taiwan.; 3 Department of Mechanical Engineering, National Chung Cheng University, Chia-Yi 62102, Taiwan.; 4 Department of Gastroenterology, Kaohsiung Armed Forces General Hospital, 2, Zhongzheng 1st.Rd., Lingya District, Kaohsiung City 80284, Taiwan.; 5 Department of Nursing, Tajen University, 20, Weixin Rd., Yanpu Township, Pingtung County 90741, Taiwan.; 6 Hitspectra Intelligent Technology Co., Ltd., Kaohsiung City 80661, Taiwan.

**Keywords:** esophageal Cancer, hyperspectral imaging, SAVE, dysplasia, SSD, YOLOv5, YOLOv8, narrow-band imaging, white light imaging

## Abstract

Band selection is a common approach to reduce the data dimensionality of hyperspectral imagery. It extracts several bands of importance in some sense by taking advantage of high spectral correlation. In medical imaging, narrow-band imaging (NBI) is an imaging technique for endoscopic diagnostic medical tests, where light of specific blue and green wavelengths is used to enhance the detail of certain aspects of the surface of the mucosa. A special filter is electronically activated by a switch in the endoscope leading to the use of ambient light of wavelengths of 415 nm (blue) and 540 nm (green). Because the peak light absorption of hemoglobin occurs at these wavelengths, blood vessels will appear very dark, allowing for their improved visibility and in the improved identification of other surface structures. NBI when compared with the white-light imaging (WLI) have proven to have better precision when combined with computer-aided diagnosis (CAD, Intespec C, Hitspectra Intelligent Technology Co., Kaohsiung, Taiwan) in detecting cancerous images. NBI endoscopes are specialized equipment that may not be widely available in all healthcare settings. By leveraging existing WLI endoscopic systems and developing algorithms to simulate NBI imaging, healthcare facilities can achieve similar di-agnostic capabilities without the need for additional costly equipment. Therefore, in this study, algorithm known as the SAVE (spectrum-aided visual enhancer) has been developed which can simulate NBI from the WLI images through an intelligent band-selective hyperspectral imaging for Olympus endoscope. The results suggested that the SAVE-NBI images had a better precision and F1-score than the WLI images.

## Introduction

A hyperspectral imager captures intricate spectral data by utilizing hundreds of narrow bands. 1. These narrow-band images (NBI) can be in the ultra-violet (UV) 2, visible (VIS), near infra-red (NIR) 3, and mid infra-red (MIR) bands 4. NBI are currently utilized in various applications such as 5, 6, medical imaging 7-9, remote sensing 10, 11, military 12, 13, agriculture 14, 15 to include a few. Hyperspectral imaging (HSI) is a developing technology that combines traditional imaging and spectroscopy to capture spatial and spectral data from an object 16. Hyperspectral images are composed of hypercubes, which are three-dimensional data blocks consisting of two spatial dimensions and one wavelength dimension across the electromagnetic spectrum 17. HSI generates a substantial amount of data because it has a higher number of bands 18. Redundancy may exist between bands, with some bands potentially having less discriminative information, leading to increased demands on storage space, computational load, and communication bandwidth, which are not conducive to real-time applications 19. It is beneficial to eliminate bands that provide minimal or no distinguishing information.

Band selection is the process of choosing a limited number of hyperspectral bands to eliminate spectral duplication and decrease computational expenses, while still maintaining the important spectral details of objects on the ground 20. Dimensionality reduction is a crucial preprocessing step for HSI analysis. Its goal is to eliminate spectral redundancy while retaining essential information for future use 21, 22. Hyperspectral dimensionality reduction can be accomplished by band selection or feature extraction 23. Many feature extraction methods combine all original bands linearly, which can complicate result interpretation if the chosen criterion is not suitable 24. However, this study focuses on the band selection. Narrow band imaging (NBI) and Multi-Band Imaging (MBI) are endoscopic imaging techniques that provide real-time visualization of the vascular network and surface texture of the mucosa to aid in tissue characterization, differentiation, and diagnosis 25. NBI's fundamental principle relies on the absorption of hemoglobin and the wavelength-specific traits of tissue optical properties 26. Hemoglobin exhibits absorption peaks at 415 nm (blue) and 540 nm (green) in the spectrum, whereas mucosal scattering decreases consistently as wavelength increases 27, 28. 415 nm light can enhance mucosal capillaries in superficial areas, while 540 nm light improves the visualization of vessels in deeper regions 29, 30.

Previous studies have demonstrated that utilizing NBI in conjunction with CAD yields superior outcomes compared to using white-light imaging (WLI) alone 31-34. Tatsugami *et al.* assessed the effectiveness of NBI cystoscopy in detecting bladder cancer and examined its diagnostic accuracy in cases of carcinoma *in situ* (CIS) and cases with known urine cytology results 35. Zhu *et al.*'s study demonstrated that Narrow Band Imaging (NBI) was more effective than White Light Imaging (WLI) in detecting both early and invasive lung cancer 36. Watanabe *et al.* assessed the effectiveness of laryngoscopy with NBI system for diagnosing precancerous and cancerous laryngeal lesions and concluded that NBI endoscopy is a highly promising diagnostic tool for laryngeal malignant disease 37. Olympus Medical Systems now offers a commercially available NBI system globally, which serves as an endoscopic diagnostic tool for the gastrointestinal (GI) tract 38. However, implementing NBI technology necessitates acquiring specialized endoscopes and video processors that are equipped with NBI capabilities. The high initial equipment cost and continuous maintenance expenses can be substantial, limiting accessibility in certain healthcare environments. NBI technology may not be widely accessible in all healthcare facilities, particularly in resource-constrained settings or smaller clinics that lack standardization 39. Therefore, an algorithm that is referred to as the SAVE (spectrum-aided visual enhancer, Transfer N, Hitspectra Intelligent Technology Co., Kaohsiung, Taiwan) and a band selection for esophageal cancer detection has been developed in this study. This algorithm has the capability to simulate NBI from the WLI images by utilizing an intelligent band-selective hyperspectral imaging mechanism for the Olympus endoscope.

## Material and Methods

### Dataset

**Figure 1:**
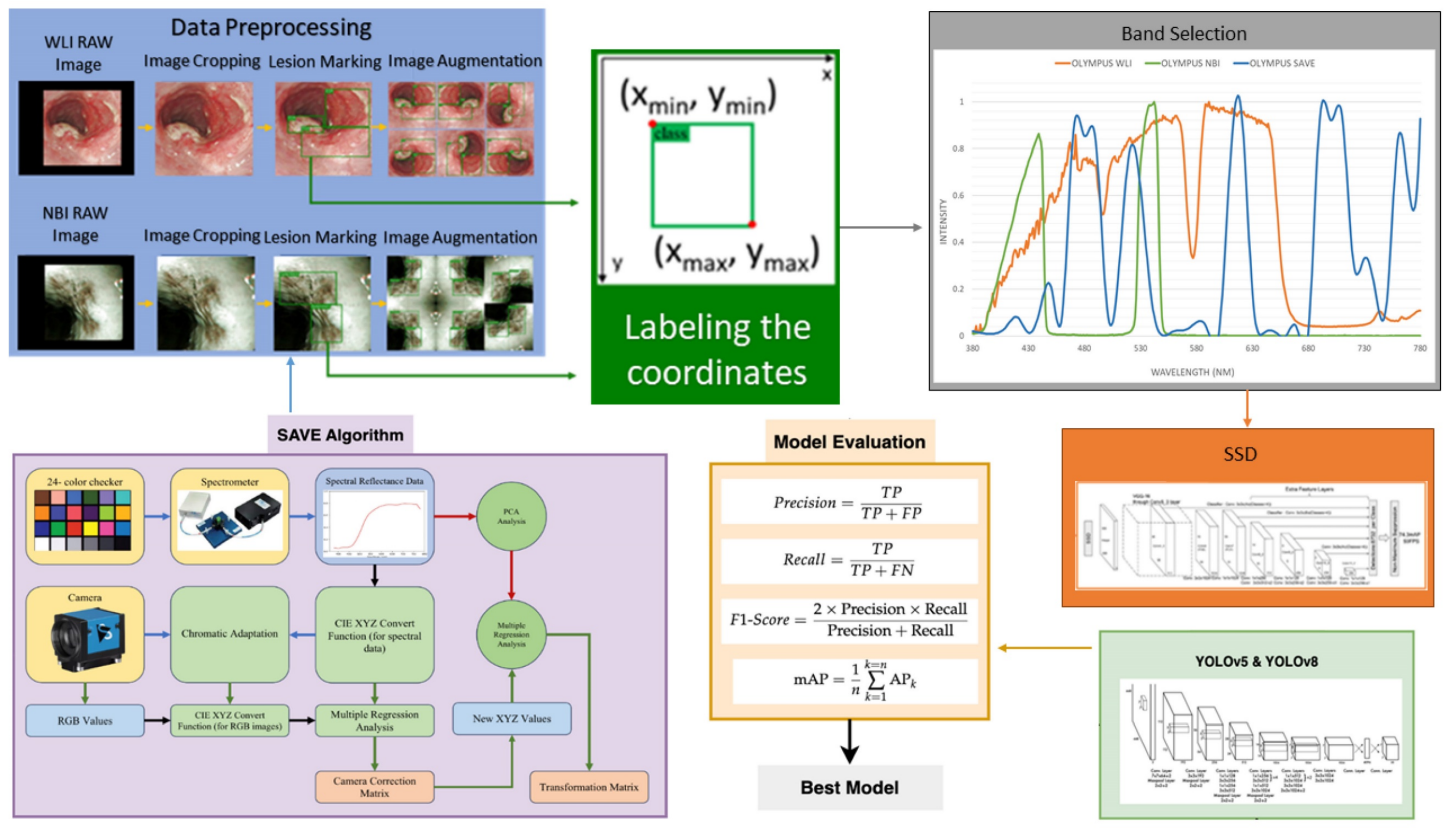
Flowchart of the Process developed in this study.

In this study a dataset consisting of 2761 WLI images, 2761 WLI images were converted to NBI images, and 935 original NBI images used were acquired with the conventional endoscope (CV-290, Olympus) for our investigation. These pictures were resized to 640×640 pixels during preprocessing to avoid possible problems such as limited computer capacity and maintain consistency in format. The process of image annotation was carried out using the widely used LabelImg software, resulting in the creation of an XML file 40. Although WLI is very effective in diagnosing esophageal cancer, its ability to diagnose dysplasia is limited. Dysplasia often has a visual resemblance to SCC, which makes it difficult to accurately identify with WLI. Thus, the use of HSI conversion methods improves the quality of the images by generating a comprehensive dataset that collects spectrum information across different wavelengths. The overall flow of the study can be seen in Figure 1. It is crucial to emphasize that the annotated dataset will be converted into HSI-NBI pictures utilizing SAVE technology throughout the project. Consequently, three datasets were obtained using WLI, NBI and HSI-NBI images. The dataset was divided into training, testing, and validation sets at a ratio of 70:15:15. Specifically, for the WLI images, 1933 images were used for training, 414 for testing, and 414 for validation. This division ensures a balanced distribution of data across the sets, enabling robust training, accurate testing, and reliable validation of the models used in the study. The WLI images were converted into SAVE images, therefore the same training, validation and testing images was used in the SAVE dataset as well. Moreover, the 935 images of NBI captured was used in the process of creating the HSI-conversion algorithm and not for the training purpose. In this study, WLI and NBI images were not captured from the same lesion. Unlike other IEE methods, such as FICE, NBI images cannot be taken simultaneously with WLI images. Therefore, WLI images were used to generate HSI-NBI images through the SAVE algorithm, and the actual NBI images served as a reference for comparison. This approach allowed us to evaluate the effectiveness of the SAVE-generated HSI-NBI images in replicating the diagnostic quality of true NBI images. To avoid data leakage, images from the same lesion were kept within a single subset (either training, testing, or validation). This ensured that the model was trained, tested, and validated on independent data, maintaining the integrity of the performance evaluation. The PyTorch deep learning framework was built on the Windows 11 operating system. The Python application was run in Jupyter Notebook, leveraging Python version 3.9.18. The training time of the three models YOLOv5, YOLOv8, and SSD models are 8 hours, 8.5, and 7 hours respectively.

### Band selection

To calculate the conversion matrix that converts the RGB image into a hyperspectral image, the spectrometer (model number QE65000 from Ocean Optics) and endoscopic camera must be calibrated. This may be done by giving the spectrometer and endoscope common color targets. This example uses the x-rite traditional 24-color checker. This color checker was selected because it has six hues of gray and most natural colors. Since endoscope RGB is sRGB, the pictures are converted to CIE 1931 XYZ. The three channels for red, green, and blue will be 0 to 255 before the conversion, then scaled down between 0 and 1. Gamma function was used to transfer color spaces. Mistakes in color separation of filters, color shift, and camera dark current will cause mistakes in this technique. The findings will be wrong due to these errors. To account for these factors, first a matrix expressing the variable *V* was calculated and performed a regression analysis to get the matrix denoting the correction coefficients *C*, as shown in equation 1.


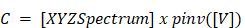

(1)

The spectrometer provides the values of *XYZ_Spectrum_*. The XYZ color gamut's *Y* value is proportional to brightness (0-100) which can be obtained from the light-source spectrum and XYZ color matching function. The *XYZ_Spectrum_* begins with the luminance ratio (*k*), which is calculated from this value after normalization. Equation 2 yields the value of the *XYZ_Correct_*. It was also found that the *XYZ_Spectrum_* and *XYZ_Correct_* had 0.5355 RMSE differences making this calibration is necessary. *R_Spectrum_* and *XYZ_Correct_*, the spectrometer reflection and camera corrected spectrum, are assessed. Principle component analysis (PCA) and multiple regression analysis are used to get the conversion matrix (*M*) and the principal components with the highest significance (PS). *V_Color_* was employed in the regression analysis of *XYZ_Correct_* and PS since it covers most X, Y, and Z combinations. *M* may be calculated from equation 3, and equation 4 generates the analog spectrum.


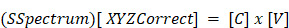

(2)


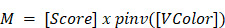

(3)


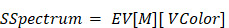

(4)

When comparing the final reflection spectrum to the real data, each block's average RMSE was 0.0532, which is insignificant. This was found by comparing the actual and final reflection spectrum values. Thus, using the approach described above, any RGB picture may be converted into a hyperspectral image with a resolution of up to 1 nm and a reflection spectrum identified. The HSI images replicated from the WLI images are named as SAVE (Spectrum-Aided Visual Enhancer) images.

**Figure 2:**
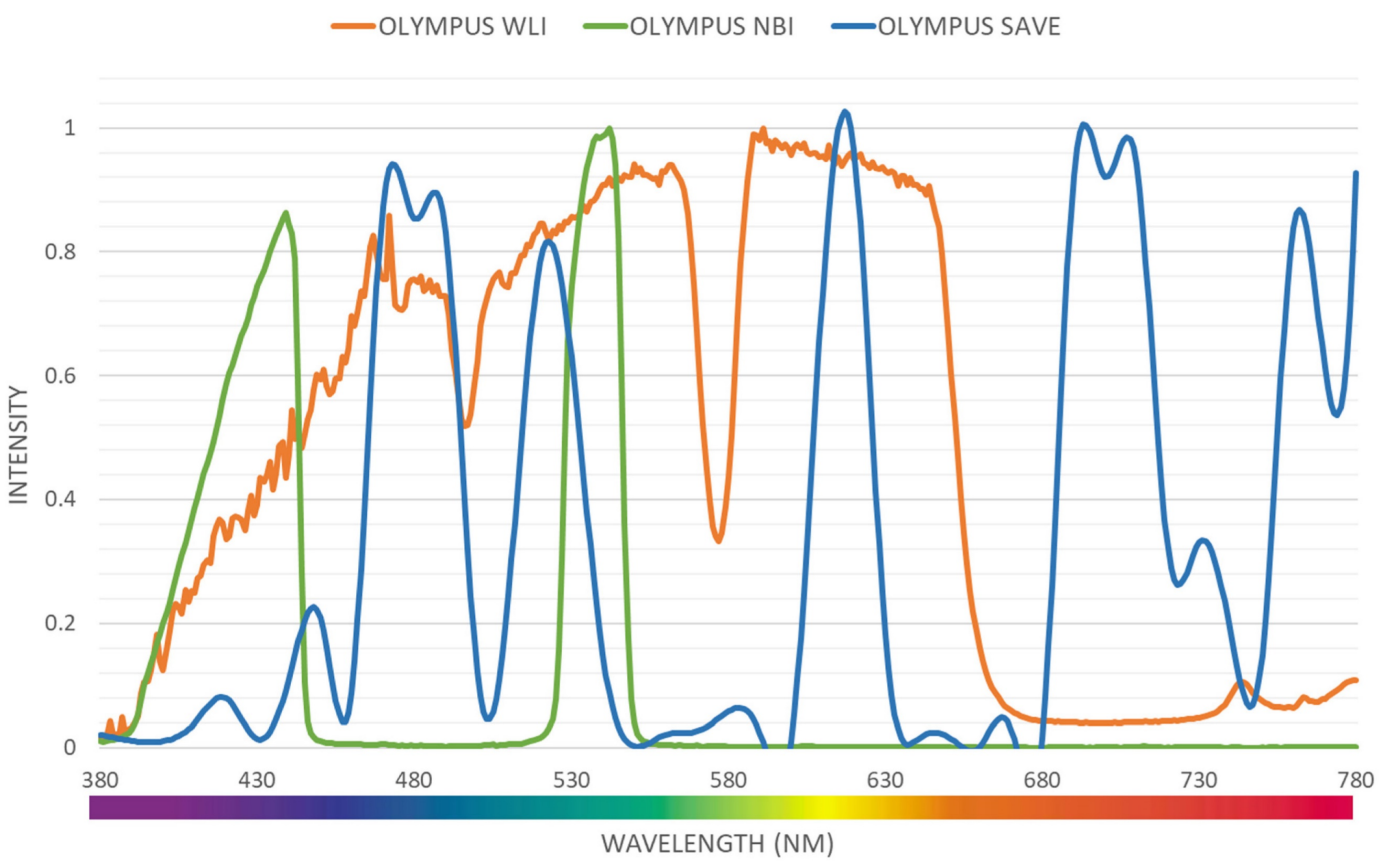
Light intensity of different wavelength for Olympus WLI, Olympus NBI and SAVE images.

Even though a normal WLI image can be converted to an HSI image, band selection is essential to create a NBI from the HSI images. Luckily, for Olympus endoscope there is a reference NBI capture mode through which an algorithm developed can be compared. Therefore, for Olympus endoscope there is a reference NBI capture mode through which an algorithm developed can be compared. Three factors will affect the color difference between the real NBI and simulated enhanced SAVE images. These factors correspond to the light spectrum, the color-matching function, reflection spectrum. At first, the CIEDE 2000 color difference between the HSI images and the Olympus endoscope was found. A similar CIEDE 2000 color difference can be found between the HSI images of the capsule endoscopes and other traditional endoscopes with the Olympus NBI image can be found to generate SAVE images for the respective endoscopes making it a versatile application. As shown in Figure 2 the spectrum of the light used in the Olympus WLI endoscope and Olympus NBI images are different. Here, Olympus claims to use 420 nm and 540 nm wavelength filters to achieve the NBI images. However, when NBI images of the Olympus are seen, there seem to be many more bands to make the NBI images more realistic. However, 415 and 540 nm are the peak light absorption of hemoglobin occurs, the lighting spectrum must also be calibrated. It was done using the Cauchy-Lorentz visiting distribution as shown in equation 5.


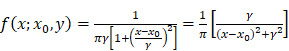

(5)

A 24-color checker is again used for calibrating the real Olympus NBI image with the enhanced SAVE images. For optimizing the lighting spectrum, the dual annealing optimization function is used. This aleatory technique, which was evolved from generalized simulated annealing algorithm which blends the simplified classical simulated annealing (CSA) and fast simulated annealing (FSA), along with an approach for executing a local search based on only certain parameters. Previously the same algorithm has been used for global optimization. The average standard CIEDE 2000 color difference between the 24 color is 5.36 which is negligible. Even though the peak absorption wavelength of hemoglobin is 415 and 540 nm, the real NBI image captured by the Olympus endoscope has not only green and blue but shades of brown colors also which corresponds to the wavelength of 650 nm. Therefore, it can be considered that there is a subtle image post-processing which makes the NBI videos more realistic. Hence in this study, apart from 415 and 540 nm three other regions in the wavelength of 480, 600, 700 and 780 nm have also light spectrum as shown in Figure 2.

### Machine Learning Model

#### YOLO

The research used the YOLOv5 D-CNN model developed by Ultralytics 7. This model employs a grid-based approach, where the input picture is divided into a grid of size SxS. Once an item's precise position is determined inside a grid, the grid assumes the task of detecting the object. The offset is derived by the difference in aspect ratio between the preceding box (Anchor) and the computed value. The grid is classified into either the background or the item category by using a pre-established threshold. Additionally, the technique involves including two adjacent grids located at the middle of the physical frame and aligned with the grid. Ultimately, the combined function of the three grids is crucial in deciding the detection outcome of the present frame. The YOLOv5 model utilizes the displacement of the center position (tx, ty) and the proportionate width and height (tw, th) of the forecasted frame in relation to the preceding frame in order to generate predictions. The confidence score and classification probability are calculated simultaneously. The final forecast box probability is determined by performing screening using the IoU threshold and the highest value of NMS. The YOLOv5 neural network architecture consists of four main components: input, feature extraction, feature analysis network, and prediction result output. The input stage employs three techniques to enhance characteristics: mosaic data augmentation, adaptive anchor box computation, and adaptive image scaling. Mosaic data augmentation improves the model's ability to precisely detect and localize small objects inside pictures. Prior to being inputted into the main network, the input data is subjected to a segmentation procedure. The focus structure enhances the channel count of the original three-channel pictures to 12. The CSP architecture reduces parameters and model size by improving the network structure design. The neck module enhances the integration of network features by using the FPN+PAN architecture and integrating bigger feature maps to compensate for the information degradation at the upper levels of the feature pyramid. The YOLOv5 model utilizes the CIOU_Loss function to evaluate the recognition loss of the identified target rectangle during the prediction process. Ultralytics released YOLOv8 in January 2023 41. It is the latest version of the YOLO real-time object recognition models and is much better than the ones that came before it 42. YOLOv8 improves efficiency by lowering box estimates and speeding up the non-maximum suppression (NMS) process by using an anchor-free design 43. This is especially helpful when dealing with items that have different forms and aspect ratios. YOLOv8's core is similar to YOLOv5, and it includes the C2f module, which was based on the ELAN idea from YOLOv7. This improves information about gradient flow while keeping the structure light. The SPPF element is kept at the end of the backbone to ensure accuracy on all sizes. YOLOv8 uses the PAN-FPN feature fusion method in the neck section and adds multiple C2f modules as well as a separated head structure that was based on YOLOx. By mixing confidence and regression boxes, they reach a new level of accuracy. Because YOLOv8 is so flexible, it can handle all YOLO versions and switch between them easily, so it can work with a lot of different hardware systems 44. The network architecture is composed of convolution, batch normalization, and SiLu activation functions.

#### SSD

The Single-shot multi-box detector (SSD) is a target detector that can recognize many categories in a single shot built using CNN 45, 46. The SSD used in this research employed a detection framework based on the VGG-16 network. VGG-16 is a deep learning model that has 16 layers, including 13 convolutional layers and 3 fully connected layers. The prediction models' findings were displayed using two methods: displaying the prediction frame and ground truth visually, together with the level of confidence in the forecast. The level of correlation between the predicted outcomes and the annotated location was assessed in relation to the impact of the prediction model on the predicted outcomes. Subsequently, the models' predictive capabilities were assessed using several assessment metrics, including sensitivity, specificity, F1-score, and mean average precision (mAP). The sensitivity of the model reflects its ability to accurately identify symptoms associated with esophageal cancer. The F1-score is a mathematical average that combines precision and recall, providing a reliable measure of the model's performance. mAP, short for mean Average Precision, is a widely used metric for assessing object detection. It quantifies the entire performance of a prediction model by considering both sensitivity and accuracy.

## Results

This work employs a binary classification methodology to identify esophageal cancer by using advanced deep learning algorithms, including YOLOv5, YOLOv8, and SSD. Rather than categorizing images into many classifications indicating various forms of esophageal cancer, the images were grouped into two unique categories: "Cancerous Images" and "Non-Cancerous Images." To enhance implementation and interpretation, we streamlined the categorization job. This technique simplifies the diagnostic procedure, enhancing its accessibility for professionals. The main goal of our study is to distinguish between malignant and benign disorders in the esophagus, which is of great importance in clinical practice. By concentrating on this dichotomous categorization, we provide practical information for healthcare professionals. Acquiring labeled data for many categories of esophageal cancer in this specific scenario is difficult, resulting in a shortage of data or an imbalance in class distribution. By simplifying the problem to a binary classification job, we address these difficulties and improve the model's performance. Binary classification models include intrinsic interpretability in contrast to approaches that involve several classes. The openness is crucial in medical applications, as comprehending the foundation of forecasts is vital for establishing confidence and acceptance. An accurate binary classifier that detects malignant pictures effectively functions as a decision support tool for physicians. This aids in selecting situations for further assessment or action, eventually enhancing patient outcomes. The results of the three models are shown in Table 1.

**Figure 3:**
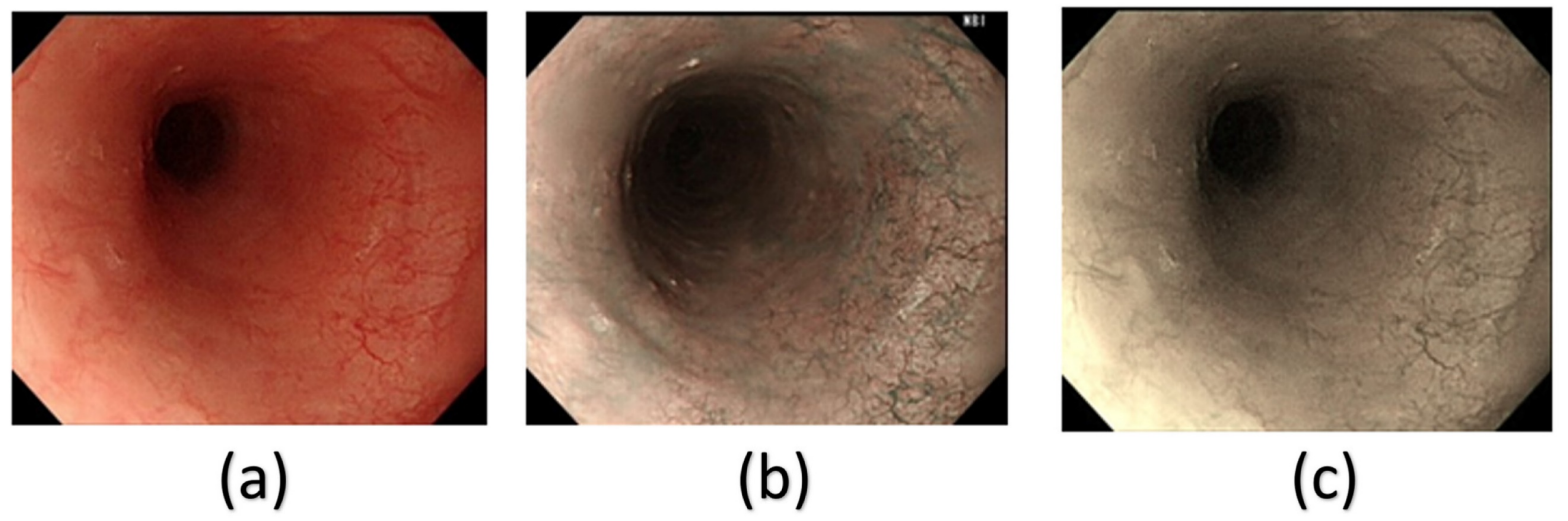
Example of a (a) WLI image from Olympus endoscope, (b) original NBI captured by Olympus endoscope and (c) the enhanced SAVE images obtained from the WLI image.

From Table 1, it can be clearly seen that the SSD model had a much better precision, recall and mAP50 in the HSI spectrum. However, we can clearly, infer from Table 1 that in all the different architectures, the HSI spectrum models performed better than the WLI-RGB models. In this study, the SSD model performed much better than the YOLOv5 and YOLOv8 models. YOLOv5 and YOLOv8 had a precision of 76.4% and 74% in the non-cancerous images in the WLI-RGB model while the HSI spectrum had a precision of 77.1% and 74.8% showing a minimal increase if using the HSI model. However, in the case of cancerous images YOLOv5 model showed an increase of 5.05% while YOLOv8 model showed an increase of 7.45% in precision. While the SSD model showed an increase of 6.4% increase in the non-cancerous images while cancerous images showed an increase of 12.75% increase in precision. Figure 3 shows the comparison between the WLI image captured by the Olympus endoscope, a similar NBI image also captured by the Olympus endoscope and the SAVE reproduced image based on the WLI images. In the WLI images, the CIE DE76, CIE DE1994, and CIE DE2000 color differences were 65.802, 9.927, and 12.7924, respectively. In comparison, the SAVE images showed significantly larger color differences, with CIE DE76, CIE DE1994, and CIE DE2000 values of 112.36, 54.85, and 40.71, respectively, between normal and cancerous tissue regions. This demonstrates that the SAVE images provide a more pronounced contrast between the lesion and surrounding tissue, making it easier to visually differentiate cancerous regions.

## Discussion

SSD surpasses YOLO in this situation due to its use of feature maps at multiple scales, allowing for more effective detection of objects of varying sizes. On the contrary, YOLO operates solely on one level, which may result in limitations. SSD has a briefer training duration than YOLO models. The efficiency of SSD is attributed to its simple design and its one-shot detection technique, which examines the entire image in one go. The YOLO model is efficient in performing inference but requires longer training times due to its complex design. On the contrary, YOLO typically has faster inference rates than SSD because it can analyze the entire image at once, whereas SSD analyzes the image multiple times at different sizes, leading to increased computational complexity. Therefore, the suitability of YOLO designs and SSD model depends on the criteria mentioned above. Moreover, the recall values demonstrate significantly lower values in comparison to the accuracy values. Recall, also known as sensitivity, measures the proportion of correctly identified positive instances out of all actual positive cases by the model. To calculate, divide the number of true positives by the sum of true positives and false negatives. The dataset's significant imbalance, with a large number of negative instances compared to positive ones, can result in an overestimation of accuracy. This occurs because when the model generates a few positive predictions, they are highly probable to be correct, leading to a high accuracy level. The recall was compromised because the model could not accurately identify many positive instances among the majority of negative cases. In the field of medical diagnostics, accuracy should be prioritized over recall. Models were adjusted to reduce the number of false positive predictions, even at the risk of missing some true positive predictions. This compromise led to increased accuracy but decreased comprehensiveness. Regulatory regulations and clinical approval procedures heavily influence the use of models in medical applications. Choosing between YOLO and SSD may also be affected by factors like interpretability, explainability, and validation requirements set by regulatory bodies or clinical standards. Utilizing SSD's multi-scale feature maps and anchor boxes can improve object identification by offering greater clarity, thereby enhancing model interpretability and ensuring regulatory compliance in specific scenarios.

## Conclusion

Band selection is a typical hyperspectral images data reduction method. It uses strong spectral correlation to extract numerous important bands. For endoscopic diagnostic medical testing, NBI uses blue and green light to increase mucosa surface detail. A switch in the endoscope activates a filter to utilize ambient light at 415 nm and 540 nm. Because hemoglobin absorbs the most light at these wavelengths, blood vessels look black, improving visibility and surface structure identification. NBI endoscopes are specialized and not ubiquitous in healthcare. Healthcare institutions may attain comparable diagnostic capabilities without expensive equipment by using WLI endoscopic devices and creating algorithms to replicate NBI imaging. This work created the SAVE program to mimic NBI from WLI pictures using intelligent band-selective hyperspectral imaging for Olympus endoscopes. The findings showed that SAVE-NBI photos had superior accuracy and F1-score than WLI images. The SSD model outperformed YOLOv5 and YOLOv8 in this investigation. YOLOv5 and YOLOv8 had 76.4% and 74% accuracy in non-cancerous photos in the WLI-RGB model, whereas the HSI spectrum had 77.1% and 74.8%, a modest gain. YOLOv5 and v8 models increased accuracy by 5.05% and 7.45%, respectively, for malignant pictures. The SSD model increased accuracy by 6.4% in non-cancerous pictures and 12.75% in malignant images.

## Supplementary Material

Supplementary methods, figures and information.

## Figures and Tables

**Table 1:** Precision, recall, F1-score and mAP50 of YOLOv5, YOLOv8 and SSD models.

Framework	Model		Metrics		
YOLOv5	WLI RGB	Precision	Recall	F1-Score	mAP50
	Non-Cancers Images	76.4%	64.9%	70.2%	68.3%
	Cancerous Images	70.7%	55.85%	61.5%	60.9%
	HSI Spectrum	Precision	Recall	F1-Score	mAP50
	Non-Cancers Images	77.1%	63.4%	69.6%	67.5%
	Cancerous Images	75.75%	56.2%	64.35%	60%
YOLOv8	WLI RGB	Precision	Recall	F1-Score	mAP50
	Non-Cancers Images	74%	65.1%	69.3%	68.2%
	Cancerous Images	68%	55%	60%	58.8%
	HSI Spectrum	Precision	Recall	F1-Score	mAP50
	Non-Cancers Images	74.8%	61.4%	67.4%	65.6%
	Cancerous Images	76.45%	51.7%	61.3%	57.6%
SSD	WLI RGB	Precision	Recall	F1-Score	mAP50
	Non-Cancers Images	84.5%	95.533%	88%	75.3%
	Cancerous Images	68%	55%	60%	59%
	HSI Spectrum	Precision	Recall	F1-Score	mAP50
	Non-Cancers Images	90.9%	71.344%	79.9%	78.9%
	Cancerous Images	89.75%	%	89.25%	86.05%
