# Supplementary material for: Evaluation of Band Selection for Spectrum-Aided Visual Enhancer (SAVE) for Esophageal Cancer Detection
Source: J Cancer. 2025 Jan 1;16(2):470–8. doi: 10.7150/jca.102759 (PMC11685682; doi:10.7150/jca.102759)
Supplement: Supplementary file 1 — Supplementary methods, figures and information. [file jcav16p0470s1.pdf]

Supplementary Material

1 Machine Learning Models

The YOLOv5 architecture adopts a single-stage detector design, featuring three integral components: Backbone, Neck, and Head, each playing a crucial role in the process of effective object detection. Figure S1 visually displays the YOLOv5 architecture [1]. Serving as the initial stage, the Backbone, named CSP-Darknet53, is responsible for extracting intricate feature representations from input images. This step ensures that the model captures nuanced details essential for precise object detection. Incorporating both SPP (Spatial Pyramid Pooling) and PANet (Path Aggregation Network), the Neck component of YOLOv5 enhances the architecture's adaptability. SPP facilitates the extraction of feature pyramids, allowing the model to generalize effectively across objects of varying sizes and scales. Concurrently, PANet contributes to improved feature representation by aggregating information from different network paths. Together, these components empower YOLOv5 with the flexibility required for detecting objects across diverse scenarios. The final component, the Head, marks the culmination of the architecture, where anchor boxes are applied to feature maps generated by the Neck. These anchor boxes serve as reference points for predicting the ultimate output, encompassing class labels, objectness scores, and bounding box coordinates. The strategic use of anchor boxes facilitates precise object localization and robust classification, underscoring YOLOv5's overall accuracy in object detection.

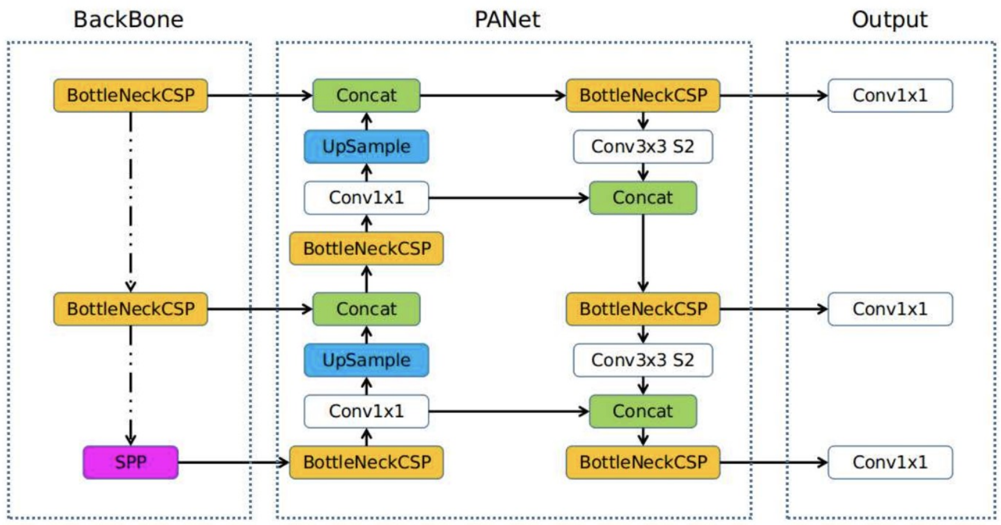

Supplementary Figure 1. YOLOv5 Architecture [1]

As per the official documentation, YOLOv8 stands as the most recent iteration of the YOLO object detection and image segmentation model created by Ultralytics. Representing a state-of-the-art model, YOLOv8 builds upon the accomplishments of its predecessors, introducing novel features and enhancements to elevate both performance and adaptability. YOLOv8 architecture introduces several key enhancements, including mosaic data augmentation, anchor-free detection, a C2f module in the backbone, a decoupled head, and a modified loss function. Mosaic data augmentation combines four images to provide contextual information, optimizing model training. Anchor-free detection replaces predefined anchors, improving generalization and expediting Non-max Suppression. The C2f module in the backbone concatenates bottleneck module outputs, enhancing computational efficiency. The decoupled head separates classification and regression tasks, potentially causing misalignment. To address this, a task alignment score is introduced, guiding the model in selecting and optimizing positive samples using BCE, CIoU, and DFL loss functions. BCE ensures label prediction accuracy, CIoU refines bounding box positioning, and DFL optimizes boundary distribution for improved overall performance. Figure S4 illustrates the YOLOv8 architecture, highlighting its features for enhanced object detection [2].

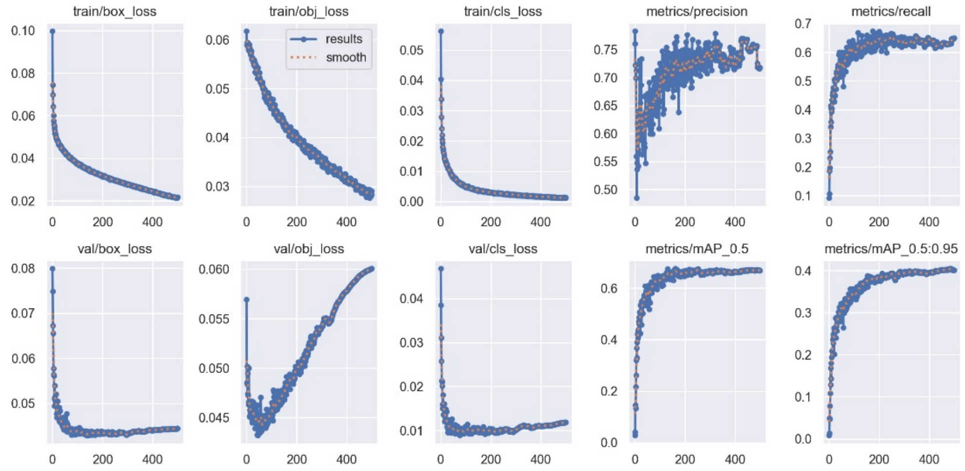

Supplementary Figure 2. YOLOv5 White-Light image training set and validation set loss functions and convergence of precision, recall, and mean precision

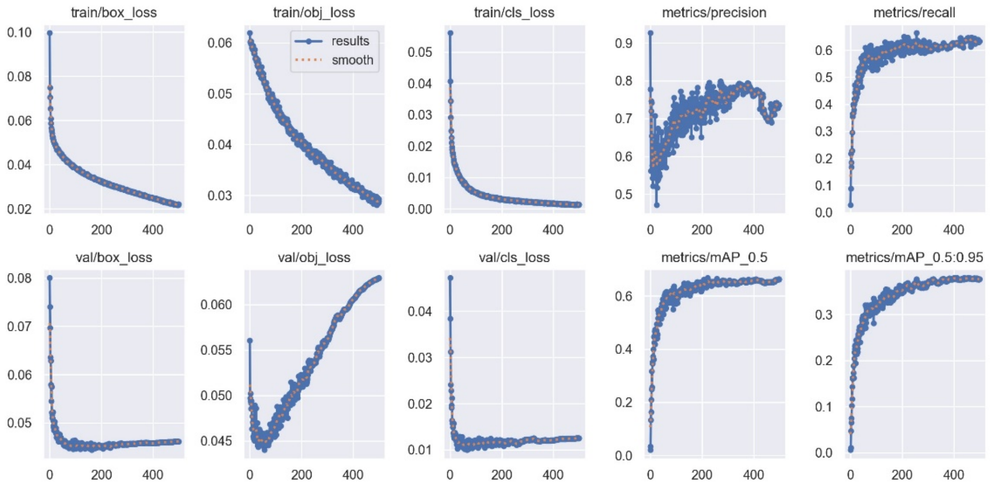

Supplementary Figure 3. YOLOv5 Hyperspectral image training set and validation set loss functions and convergence of precision, recall, and mean precision

In 2015, the Single Shot Multi-Box Detector (SSD) was proposed. It is constructed by a Convolutional Neural Network (CNN) and is a fast single-shot multi-category target detector. Figure S1 is the architecture diagram of SSD. The SSD used in this study is a detection architecture based on the VGG-16-Atrous1 network. Vgg-16 is composed of 16 hidden layers with 13 convolutional layers and 3 fully connected layers. SSD uses a feature extraction network, removes the two fully connected layers of fc6 and fc7, and adds a pyramidal feature hierarchy to detect feature maps of different sizes: large detection of small objects, small detection of large objects. In this way, the detection of small targets is strengthened, and the mechanism of anchors is used. Default boxes of a certain size are preset in advance, which can reduce the difficulty of training. Using the pyramidal feature hierarchy, has 6 layers of convolutional layers, so the size of the feature map is also reduced, and the detection scale is gradually reduced. The advantage is that it can be used for different sizes. The large feature map can be used to detect small objects, while the small feature map can be used to detect large objects, as shown in Figure S3. SSD will generate feature maps of different scales, and two 3x3 CNNs will be connected behind each feature map, one will output the location and the other will output the confidence, so as to obtain a series of prediction results. Each small cell is called a feature map cell, and the preset default frame size and the relative position of the cell are fixed. The predicted frame is not an absolute position but is relative to the real frame offset. Therefore, combined with the previous two sections, the multiple feature maps generated by the pyramid structure will predict the object according to the preset default frame size, and generate a series of different types of position information and corresponding confidence.

2 Equation of Object Detection Indicators

In esophageal cancer detection, precision, recall, F1-score, mean Average Precision (mAP) and confusion metrics are key metrics for evaluating tailored object detection models [3]. Precision emphasizes the accuracy of positive predictions, ensuring alignment with actual symptoms. High precision implies reliable identification of true positives while minimizing false positives in esophageal cancer symptoms.

$$Precision = \mathrm{TP}/\ (TP + FP) \quad \text{(S1)}$$

Recall is crucial, measuring the model's sensitivity to capture all relevant cancer symptoms. High recall indicates effective identification, minimizing oversight.

$$Recall = \mathrm{TP}/\ (TP + FN) \quad \text{(S2)}$$

The F1-score, a harmonic mean of precision and recall, is vital, offering a balanced assessment of overall accuracy in esophageal cancer detection.

$$F1\text{-}Score = (2 \times \mathrm{Precision} \times \mathrm{Recall})/\ (\mathrm{Precision} + \mathrm{Recall}) \quad \text{(S3)}$$

mAP (mean Average Precision), specialized for esophageal cancer, considers precision-recall curves for each symptom, providing nuanced evaluation across manifestations. This metric calculates the average precision (AP_k) for each class (k), where n represents the number of classes. The average precision of each class is then averaged to compute the mean Average Precision.

$$\mathrm{mAP} = \frac{1}{n}\sum_{k=1}^{k=n} \mathrm{AP}_k \quad \text{(S4)}$$
